# Supplementary material for: Perceptions, experiences, barriers, facilitators, learning outcomes, and modes of assessment of digital clinical placements for pre-registration physiotherapy students internationally: a systematic review protocol
Source: PLoS One. 2025 Feb 20;20(2):e0319024. doi: 10.1371/journal.pone.0319024 (PMC11841889; doi:10.1371/journal.pone.0319024)
Supplement: S1 Appendix — (PDF) [file pone.0319024.s002.pdf]

Appendix A: MEDLINE OVID Search Strategy

**1**	digit*.tw,kf.
**2**	cyber*.tw,kf.
**3**	distance*.tw,kf.
**4**	(e?care or e?consultation* or e?health or e?healthcare or e?medicine or e?monitor* or e?service* or e?visit*).tw,kf.
**5**	(electronic and (care or consultation* or health or healthcare or medicine or monitor* or service* or visit*)).tw,kf.
**6**	mobile*.tw,kf.
**7**	online*.tw,kf.
**8**	remote*.tw,kf.
**9**	tele*.tw,kf.
**10**	videoconferenc*.tw,kf.
**11**	virtual*.tw,kf.
**12**	web-based*.tw,kf.
**13**	1 or 2 or 3 or 4 or 5 or 6 or 7 or 8 or 9 or 10 or 11 or 12
**14**	exp Telemedicine/
**15**	13 or 14
**16**	physi* therap*.tw,kf.
**17**	physiotherap*.tw,kf.
**18**	16 or 17
**19**	exp Physical Therapist/
**20**	18 or 19
**21**	pre-registration*.tw,kf.
**22**	apprentice*.tw,kf.
**23**	candidate*.tw,kf.
**24**	entry-level*.tw,kf.
**25**	intern*.tw,kf.
**26**	learner*.tw,kf.
**27**	pre-licen*.tw,kf.
**28**	pre-professional*.tw,kf.
**29**	probationer*.tw,kf.
**30**	resident*.tw,kf.
**31**	school-based.tw,kf.
**32**	student*.tw,kf.
**33**	trainee*.tw,kf.
**34**	21 or 22 or 23 or 24 or 25 or 26 or 27 or 28 or 29 or 30 or 31 or 32 or 33
**35**	15 and 20 and 34
