# Supplementary material for: Perceptions, experiences, barriers, facilitators, learning outcomes, and modes of assessment of digital clinical placements for pre-registration physiotherapy students internationally: a systematic review protocol
Source: PLoS One. 2025 Feb 20;20(2):e0319024. doi: 10.1371/journal.pone.0319024 (PMC11841889; doi:10.1371/journal.pone.0319024)
Supplement: Attachment — Submitted filename: Response to Reviewers.docx [file pone.0319024.s003.docx]

Dear Editor and reviewers,

On behalf of the research team, thank you for taking the time to review our manuscript. Please find below our responses to your feedback in ‘Review Comments to the Author’.

**Feedback from reviewer 1**

**1.** I appreciated reading an entire section devoted to the definition of digital clinical placements. As you acknowledge, the diversity of definitions within existing literature indicates the need for clarity, at least how it pertains to the current work. However, I wonder if you could tighten your current approach, so that a single statement captures the essence of this concept rather than the definition being spread across three individual statements. For example, perhaps something like “healthcare delivered a/synchronously by trainees under the supervision of registered professionals to human patients entirely or partially via information and communication technology” might work well. FYI, this paper by Podsakoff and colleagues (<https://journals.sagepub.com/doi/full/10.1177/1094428115624965>) is my ‘go to’ reference when it comes to thinking about high-quality concept definitions.

**Response:**

Thank you for sharing this “go to” reference, it was an insightful read. Please see lines 92-103 in the ‘Revised Manuscript with Track Changes’ version and lines 91-99 in the ‘Manuscript’ version for how your feedback was implemented and amended to ensure consistency with the terminology throughout the paper.

**2.** I have a couple of reflections regarding the section titled ‘the state of the evidence’:
• Line 114: a single paper among how many? This context is important to help readers appreciate the essence of your reflections on their work.
• I believe you could sharpen your critical evaluation of their methodology to strengthen the gap you address here. For example, authors of this prior work may have set out to scope perspectives broadly across allied health rather than in-depth for any specific discipline. If so, your critical evaluation could be considered a strength to some extent rather than a weakness, or at least an artifact of their guiding research questions (I suspect the search strategy to capture everything across all health sciences would be complex and capture a lot of irrelevant work). Expanding the points you mention also seems important. For example, what is the value of the additional search strategies you mention (e.g., increase the likelihood that we capture most if not all relevant data)?

**Response:**

- Regarding your first point about line 114, I have added more to state the context of the total number of papers in each study. This is now in lines 121 and 122 of the ‘Revised Manuscript with Track Changes’ version and lines 115 and 116 of the ‘Manuscript’ version.
- Regarding your second point about sharpening our critical evaluation of the systematic reviews, the scope of their research was broad because of the aim of their respective research. Generally, Bridgman et al. and Romli et al. wanted to capture allied health students’ perspectives on the adoption and use of telehealth for clinical placements in the context of COVID19. In the context of our aims and objectives, these systematic reviews do not capture the physiotherapy perspective enough and that is the gap in knowledge we have identified.
- Regarding your second point about the value of additional search strategies, I have elaborated and wrote, “… by increasing the likelihood of capturing all relevant data.” Please see lines 130 and 131 in the ‘Revised Manuscript with Track Changes’ version and lines 124 and 125 in the ‘Manuscript’ version.

**3.** General writing comment: people are ‘who’ or ‘whom’ rather than ‘that’ (e.g., Table 1 “Clinical educators that have experience…”).

**Response:**

Corrected this grammar.

**4.** I preface this comment with my general impression that the QuADS and GRADE-CERQual appear reasonable enough for your purposes. Nevertheless, I also would like to add that quality appraisals and confidence in evidence for qualitative research are notoriously complex and, therefore, ‘one size fits all’ approaches are typically considered inadequate by qualitative experts (for which I am not!). As one example, Smith and McGannon eloquently explained the complexities of rigour for qualitative research in this paper (<https://www.tandfonline.com/doi/full/10.1080/1750984X.2017.1317357>). Brett Smith and others have written extensively on such issues (e.g., <https://doi.org/10.1016/j.psychsport.2009.02.006>, <https://www.tandfonline.com/doi/full/10.1080/1612197X.2019.1637363>).

**Response:**

Thank you for this feedback, we believe that for the purposes of this study, the QuADS and GRADE-CERQual will be suitable for our study. While the “one size fits all” approach may be inadequate, we believe that the QuADS and GRADE-CERQual will provide a structured approach to quality assessment that is user friendly for the authors of our study. This rationale has been added to the manuscript as seen in lines 341-343, in the ‘Revised Manuscript with Track Changes’ version, and lines 334-338 in the “Manuscript.” The papers you have referenced will be shared with all the authors to help with our reflexivity in this study.

**Feedback from reviewer 2**

**1.** In the section titled ‘the state of the evidence’, the evidence provided comes from two reviews on this topic, each of which references a single article pertaining to physiotherapy student placements. Despite this being a relatively under-explored topic of research in the physiotherapy profession, there are other journal articles (not captured by the aforementioned reviews) that could be included in this section to provide a more comprehensive overview of the topic of interest. Here are just a few examples, within physiotherapy, that could be considered for inclusion:
• <https://doi.org/10.5195/ijt.2022.6464>
• <https://doi.org/10.53300/001c.32992>
• <https://doi.org/10.53300/001c.24960>
• <https://doi.org/10.11157/fohpe.v23i3.595>
• <https://doi.org/10.1002/msc.1723>
• <https://doi.org/10.1002/hsr2.2067>

**Response:**

Thank you for your feedback. We have added evidence from the primary studies to ensure clarity and breadth. This is viewable in lines 141-192 of the ‘Revised Manuscript with Track Changes’ version and lines 135-186 of the ‘Manuscript’ version. Additionally, we offer this insight, the goal of “The state of the evidence” section was to highlight that only two systematic reviews were related to our research question – “What is the state of the evidence on digital clinical placements for pre-registered physiotherapy students?” In highlighting only two systematic reviews with low physiotherapy perspective, we highlighted the gap that exists in our current understanding, therefore identifying the need for a physiotherapy specific systematic review.

Some of the articles you recommended were not relevant to include in this section as detailed below:

- <https://doi.org/10.11157/fohpe.v23i3.595> – “Martin, R., Mandrusiak, A., Lang, R., Forbes, R., & Russell, T. (2022). A telehealth curriculum: A pre-post study of physiotherapy students’ perceived knowledge, self-efficacy and intentions for future use. *Focus on Health Professional Education: A Multi-Professional Journal*, *23*(3), 56–72.”
  - Not included because the scope of this review does not include telehealth education curriculum within the physiotherapy program. More specifically, we are trying to find evidence and research on the participation or application of digital clinical placements for pre-registration physiotherapy students. This article appears to have the aim of developing, implementing, and evaluating new telehealth curriculum within a physiotherapy program. It does not appear to be a study on the use of telehealth on a clinical placement. However, this study may be useful to discuss in relation to our findings once the review is completed.
- <https://doi.org/10.1002/msc.1723> – “Davies, L., Lawford, B., Bennell, K. L., Russell, T., & Hinman, R. S. (2023). Telehealth education and training in entry-to-practice physiotherapy programs in Australian universities: A qualitative study with university educators. *Musculoskeletal Care*, 21(2), 491–501.”
  - Not included because this article examines the integration of telehealth education in physiotherapy programs, rather than use of telehealth on clinical placements. However, this study may also be useful for discussion in relation to the findings of our study once it is completed.

**2.** In objective 1 you state ‘digital/online clinical placements’ whereas objectives 2 and 3 simply state ‘digital clinical placements’. In light of your stated definition of ‘digital clinical placements’ (lines 91-92), it would be appropriate to delete the word ‘online’ in objective 1 for consistency.

**Response:**

I have deleted the word 'online’.

**3.** Minor typographic errors:
• In Table 1 Inclusion criteria (page 14): “… using …” should read “… uses …”
• Line 294: “asses’ should read “assess”
• Table numbers appear incorrect i.e., there are two (2) Table 1’s referenced within the body of the paper (page 14 and page 26)
• In Table 1 Confidence in Cumulative Evidence (last column on page 26) and Table 2 Summary of Confidence in Cumulative Evidence (last column on page 27): “… rating give in the …” should read “… rating given in the …”

**Response:**

- Corrected “using” to “uses” in table 1.
- Corrected “asses” to “assess”.
- Table numbers have been corrected. It should have read Table 3 and Table 4.
- Corrected “give in” to “given” in Table 3 and Table 4.

**4.** Regarding data synthesis, I fully appreciate the desire to focus your meta-aggregation on commonalities among existing work. Nevertheless, as a thought-provoking comment, you also might like to consider ‘exceptions’ within the existing body of work and how they might shed light on your research questions. Phoenix and Orr wrote an excellent piece that might provide inspiration for you (<https://www.tandfonline.com/doi/full/10.1080/2159676X.2017.1282539>).

**Response:**

Thank you for this feedback too, this will be shared with all the authors of this study and we will consider the “exceptions” when we are performing our data synthesis.

**Feedback from reviewer 1 and 2**

**1.** The systematic review protocol is excellent – well done. We have a few reflections for your consideration:
• Regarding data items, you might consider adding philosophical positioning to the list, as you’ll likely access mostly qualitative studies in which this information is central to assessments of rigor. Relatedly, you also might like to add your philosophical positioning for this work so that readers can appreciate key concepts like ontology and epistemology as it relates to your efforts.
• Since you are targeting assessment modes, learning outcomes, beliefs, etc…, it is possible these aspects could be overlooked (e.g. if captured via standardized self-reports) if only accessing qualitative and mixed methods designs. As such, you could consider expanding the inclusion criteria to also include Quantitative studies. Alternatively, you may need to provide a rationale for why you are excluding quantitative only studies and edit the research objectives accordingly.
• Lines 237-240: This statement could be strengthened so that readers can appreciate exactly what you plan to do here. I’m unsure that one can collate and summarise ‘themes’ among a body of work with no interpretation on the part of analysts. You might not be interpreting the original work per se but nevertheless are ‘filtering’ that information in some way (i.e., you are an active driver rather than passive passenger). Relatedly, what exactly do you mean by a theme here?

**Response:**

Thank you for these points for us to reflect on.

- Added philosophical positioning to the list of data items and added a section on positioning in the “Methods” section.
- We had considered quantitative studies previously but decided that the scope and aim of this review did not need to include them. The addition of quantitative data would require us to create methodology for the collection, assessment, and analysis of that data, which we believed was not appropriate to the scope of identifying and synthesizing assessment modes, learning outcomes, beliefs, etc.
- Regarding lines 237-240, they are now lines 327-331 in the ‘Revised and Tracked Changes’ version and lines 321-325 in the ‘Manuscript’ version. We have clarified what is meant by themes. Additionally, we have tried to make it more clear how meta-aggregation aims to collate and summarize without re-interpretation by referring to the data synthesis section below which explains the process. now lines 333 and 334 in the ‘Revised and Tracked Changes’ version and lines 327 and 328 in the ‘Manuscript’ version.

**Edits for journal requirements**

- Level 1, 2, and 3 heading formatting per journal requirements for manuscript applied. Please see lines 1, 26, 27, 33, 46, 51, 69, 107, 194, 199, 210, 211, 240, 246, 255, 259, 264, 269, 275, 282, 285, 296, 297, 302, 316, 323, 337, 348, 365, 417, 423, 426, 441, 444, 461, and 561 in the ‘Revised Manuscript with Tracked Changes’ version and lines 25, 26, 32, 45, 50,68, 101, 188, 193, 204, 205, 234, 240, 249, 253, 258, 263, 269, 276, 279, 290, 291, 296, 310, 317, 330, 341, 358, 408, 414, 417, 432, 435, 452, and 552 in the ‘Manuscript’ version.
- Line 362 in the ‘Revised Manuscript with Tracked Changes’ version and line 355 in the ‘Manuscript’ version, ‘Figure 1’ was changed to ‘Fig 1’ per journal requirements.
- Added Figure 1 caption, “**Fig 1**: Example illustration of meta-aggregation process.” Please see line 363 in the ‘Revised Manuscript with Tracked Changes’ version and line 356 in the ‘Manuscript’ version. Figure 1 converted to a separate .tif file.
- Tables 3 and 4 added to the manuscript per journal requirements. Please see lines 406 and 413 in the ‘Revised Manuscript with Tracked Changes’ version and lines 399 and 405 in the ‘Manuscript’ version.
- Removed ‘Title’ from the titles, authors, and affiliates page per journal requirements. Please see line 1 in the ‘Revised Manuscript with Tracked Changes’ version.
- Added name of the corresponding author to the titles, authors, and affiliates page per journal requirements. Please see line 18 in the ‘Revised Manuscript with Tracked Changes’ version and line 17 in the ‘Manuscript’ version.
