# Supplementary material for: Perceptions, experiences, barriers, facilitators, learning outcomes, and modes of assessment of digital clinical placements for pre-registration physiotherapy students internationally: a systematic review protocol
Source: PLoS One. 2025 Feb 20;20(2):e0319024. doi: 10.1371/journal.pone.0319024 (PMC11841889; doi:10.1371/journal.pone.0319024)
Supplement: Attachment — Submitted filename: Reviewer response to resubmission 1.docx [file pone.0319024.s004.docx]

Reviewer Response

Many thanks for your efforts in addressing our feedback or providing rationales in cases where you disagreed with our comments. Overall, we believe the paper is strengthened after this round of reviews. We have no additional feedback. We look forward to reading the full paper with results once available.

The only one minor revision that could be considered is the addition of a word to complete the one sentence (lines 159-162) e.g., '... digital settings"
